# Quantifying microcalcification activity in the thoracic aorta

**DOI:** 10.1007/s12350-020-02458-w

**Published:** 2021-01-20

**Authors:** Alexander J. Fletcher, Maria Lembo, Jacek Kwiecinski, Maaz B. J. Syed, Jennifer Nash, Evangelos Tzolos, Rong Bing, Sebastien Cadet, Gillian MacNaught, Edwin J. R. van Beek, Alistair J. Moss, Mhairi K. Doris, Niki L. Walker, Damini Dey, Philip D. Adamson, David E. Newby, Piotr J. Slomka, Marc R. Dweck

**Affiliations:** 1 British Heart Foundation Centre for Cardiovascular Science, University of Edinburgh, Edinburgh, UK; 2 Department of Advanced Biomedical Sciences, Federico II University of Naples, Naples, Italy; 3 Department of Interventional Cardiology and Angiology, Institute of Cardiology, Warsaw, Poland; 4 Department of Vascular Surgery, Queen Elizabeth University Hospital, Glasgow, UK; 5 Department of Imaging (Division of Nuclear Cardiology), Cedars-Sinai Medical Centre, Los Angeles, USA; 6 Christchurch Heart Institute, University of Otago, Christchurch, New Zealand; 7 Edinburgh Imaging Facility, Queens Medical Research Institute, University of Edinburgh, Edinburgh, UK; 8 Scottish Adult Congenital Cardiology Service, Golden Jubilee National Hospital, Clydebank, Glasgow UK

**Keywords:** PET, Modalities, Image analysis, Technical, Molecular imaging agents, Tracers, Others, Tests

## Abstract

**Background:**

Standard methods for quantifying positron emission tomography (PET) uptake in the aorta are time consuming and may not reflect overall vessel activity. We describe aortic microcalcification activity (AMA), a novel method for quantifying ^18^F-sodium fluoride (18F-NaF) uptake in the thoracic aorta.

**Methods:**

Twenty patients underwent two hybrid ^18^F-NaF PET and computed tomography (CT) scans of the thoracic aorta less than three weeks apart. AMA, as well as maximum (TBRmax) and mean (TBRmean) tissue to background ratios, were calculated by two trained operators. Intra-observer repeatability, inter-observer repeatability and scan-rescan reproducibility were assessed. Each ^18^F-NaF quantification method was compared to validated cardiovascular risk scores.

**Results:**

Aortic microcalcification activity demonstrated excellent intra-observer (intraclass correlation coefficient 0.98) and inter-observer (intraclass correlation coefficient 0.97) repeatability with very good scan-rescan reproducibility (intraclass correlation coefficient 0.86) which were similar to previously described TBRmean and TBRmax methods. AMA analysis was much quicker to perform than standard TBR assessment (3.4min versus 15.1min, *P*<0.0001). AMA was correlated with Framingham stroke risk scores and Framingham risk score for hard cononary heart disease.

**Conclusions:**

AMA is a simple, rapid and reproducible method of quantifying global ^18^F-NaF uptake across the ascending aorta and aortic arch that correlates with cardiovascular risk scores.

**Electronic supplementary material:**

The online version of this article (10.1007/s12350-020-02458-w) contains supplementary material, which is available to authorized users.

## Introduction

Thoracic aortic calcification is an important pathological entity underlying both intimal atherosclerotic disease and medial degenerative processes.^1^ High-density macro-calcified lesions in the aortic wall can be readily identified using computed tomography (CT) and are associated with an increased risk of stroke and mortality.^1^-^4^ However, these lesions represent a late and stable stage of vascular wall pathology where active disease processes may have become quiescent.^5^ In contrast, microcalcification - typically defined as lesions <50 µm - cannot be detected on conventional imaging but can identify regions of active vascular disease.^6^,^7^^18^F-Sodium fluoride (^18^F-NaF) is a positron emitting radiotracer that allows the detection of microcalcification activity by positron emission tomography (PET),^8^ providing a marker of aortic disease which might further improve risk prediction. Indeed, coronary ^18^F-NaF PET has recently demonstrated its ability to improve risk prediction beyond that afforded by CT calcium score.^9^,^10^ Thus, there is interest in developing summary quantitative methods of measuring ^18^F-NaF uptake in the aorta, which may provide similarly important prognostic information.

**Figure 1:**
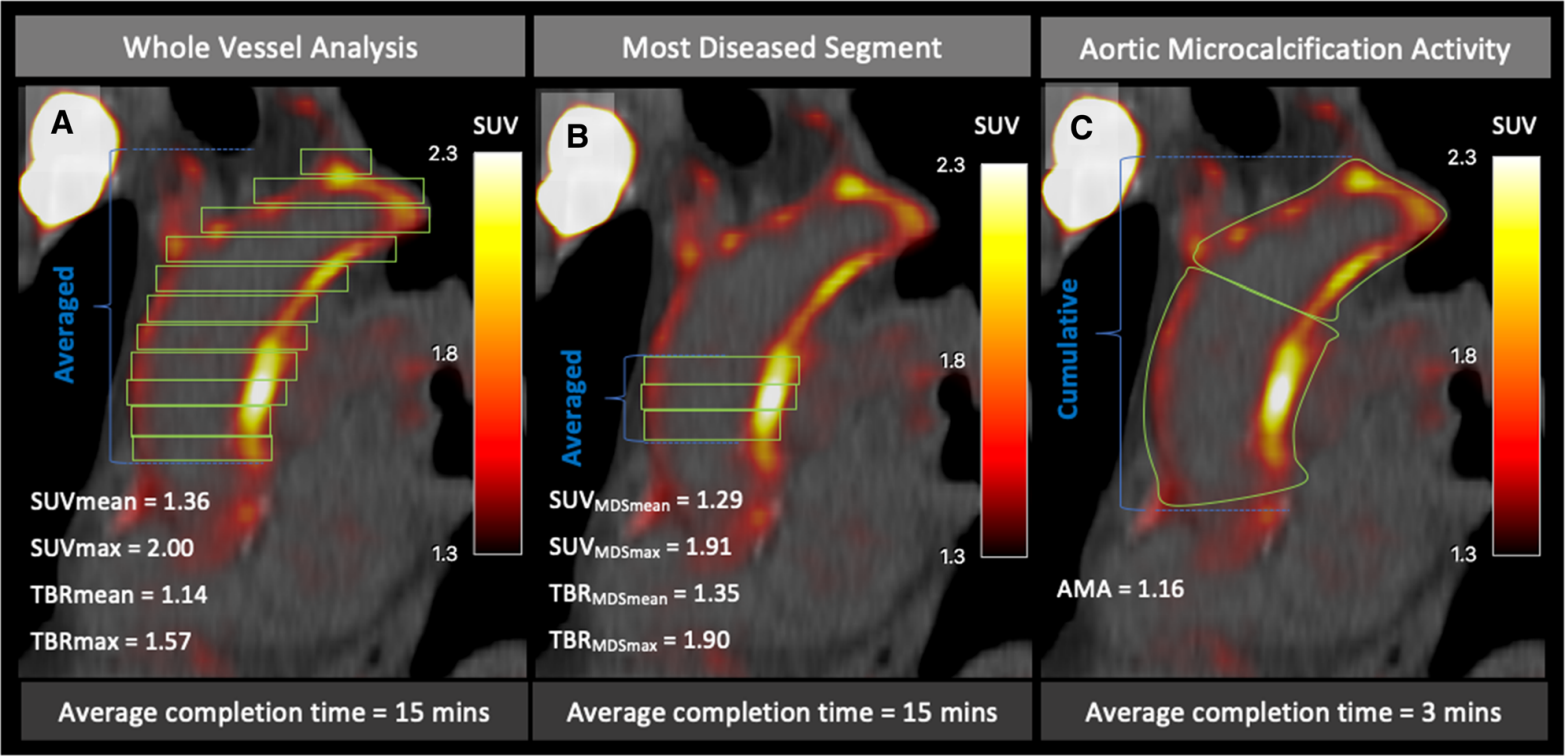
^18^F-Sodium fluoride positron emission tomography and computed tomography in a patient with marked aortic wall uptake. An illustrated representation of standard whole vessel (**A**) and most diseased segment (**B**) as well as novel aortic microcalcification (**C**) methods for quantifying uptake. Average time taken to complete each method is shown. *AMA*, aortic microcalcification activity; *Asc*, ascending aorta; *CT*, computed tomography; *PA*, pulmonary artery; *PET*, positron emission tomography; *LV*, left ventricle; *RA*, right atrium; *TBR*, tissue to background ratio

Quantification of ^18^F-sodium fluoride uptake in the thoracic aorta currently involves labour intensive analysis of multiple regions of interest across sequential axial slices and calculating mean and maximum intensity uptake values. These values are then normalized to blood pool activity to generate mean (TBRmean) and maximum (TBRmax) tissue to background ratios respectively.^11^-^13^ Typically, TBRmax values are influenced by only a small number of the most intense pixels within a volume of interest and may not accurately reflect the overall PET activity within that volume (Figure 1). A simple, robust and time-efficient technique that could provide a summary measure of PET uptake across the thoracic aorta would be a major advance. We, therefore, aimed to develop a novel method of quantifying the burden of 18F-NaF uptake across both the ascending aorta and aortic arch (aortic microcalcification activity, AMA) and to assess its repeatability, reproducibility and time-efficiency compared with current standard approaches. Finally, we provide a comparison between each method and well validated clinical risk scores for future risk of cardiovascular events.^14^,^15^

## Methods

### Study Population

Twenty patients recruited as part of the Dual anti-platelet therapy to Inhibit Atherosclerosis and Myocardial Injury in patients with Necrotic high-risk coronary plaque Disease (DIAMOND NCT02110303) study underwent two hybrid ^18^F-NaF PET-CT scans of the thoracic aorta no more than three weeks apart.^16^,^17^ Inclusion criteria for the study were patients ≥ 40 years old with angiographically confirmed multivessel coronary disease defined as epicardial vessels with >50% stenosis or having undergone previous coronary revascularisation. Exclusion criteria included acute coronary syndrome in the preceding 12 months, revascularisation in the preceding 3 months, estimated glomerular filtration rate <30 mL/min/1.73m^2^, concurrent therapy with oral anticoagulants or thienopyridine (clopidogrel or prasugrel), or known allergy to iodine contrast media. The study was approved by the local institutional review board, the Scottish Research Ethics Committee (REC reference: 14/SS/0089), the Medicines and Healthcare products Regulatory Agency, and the United Kingdom Administration of Radiation Substances Advisory Committee and written informed consent was acquired from all patients. The present work is a post-hoc analysis of this prospective randomised controlled trial.

### PET-CT Image Acquisition Protocol

All scans were performed 60 min after injection of 250 MBq of ^18^F-NaF on a hybrid PET-CT scanner (128-multidetector Biograph mCT, Siemens Medical Systems, Erlangen, Germany) at a single centre. Attenuation correction CT was performed immediately before PET data acquisition (100-120 kV, current 40-50 mA), and reconstructed at 3-mm slice thickness. The field of view incorporated the heart and whole thoracic aorta including the first branches of the head and neck vessels. PET data were acquired with ECG-gating in list-mode during a single 30-min bed position.

### Positron Emission Tomography Reconstruction

PET images were reconstructed into four cardiac phases. All PET image reconstructions were performed using the UHD algorithm which applies point-spread function and time-of-flight techniques on a 256 × 256 matrix (109 slices, slice thickness 2.027 mm) using 2 iterations, a 5-mm Gauss filter and 21 subsets. Initial analysis was performed by analysing uptake throughout the cardiac cycle (summed gate). However, we have demonstrated improved repeatability and reproducibility with correction for heart movement and blood pool clearance when assessing coronary arteries.^18^ As such, motion-corrected images of the ascending aorta and arch were also obtained applying the same custom-built algorithm as used in the coronary vessels for quantifying PET uptake (FusionQuant v1.20.05.14, Cedars-Sinai Medical Centre, Los Angeles).^19^ This motion correction function aligns the aortic uptake from all gates throughout the cardiac cycle onto the mid-diastolic gate without data loss. Finally correction for blood pool clearance and the time interval between ^18^F-NaF injection and scan acquisition were performed as described previously and applied to the background (blood-pool) activity.^20^

### Assessment of Aortic ^18^F-Sodium Fluoride Uptake

Conventional methods for assessing aortic uptake were investigated alongside AMA using FusionQuant v1.20 software as described below (Cedars-Sinai Medical Centre, Los Angeles).^21^ For all the methods, the PET signal was first carefully co-registered in 3 orthogonal planes using the non-contrast attenuation CT in all patients. Background activity in the blood pool was determined as the average standardised uptake value (SUVmean) of two 2-cm^3^ spheres of interest, one in the right atrium and one in the left atrium. The time to complete image analysis was recorded for all of the methods investigated.

#### Aortic microcalcification activity measurements

**Figure 2:**
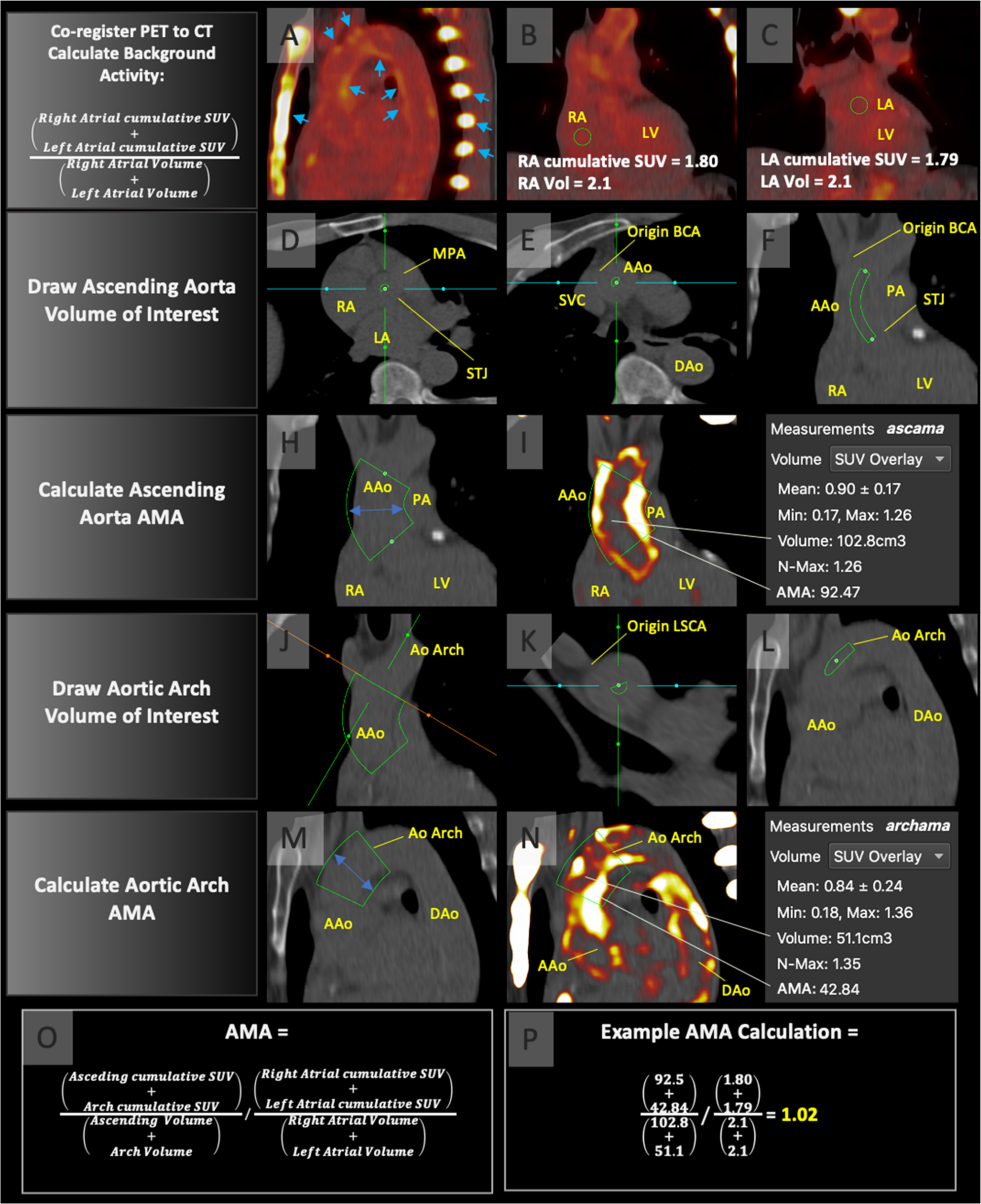
Step-by-step outline of measuring aortic microcalcification activity. (**A**) Co-register register 18F-sodium fluoride overlay to computed tomography image in three orthogonal planes using landmarks of the sternum, spine, and aortic wall (blue arrows). (**B** + **C**) Place a 2 cm^3^ region of interest in the centre of the right (**B**) and left (**C**) atrium. The background activity is the cumulative SUV per cm^3^ from the volumes of interest in the left and right atrium. (**D**, **F**) With the ^18^F-sodium fluoride overlay turned off, a centreline function is used to draw the ascending aortic volume of interest in multiplanar reconstruction images. Perpendicular to the aorta, the volume of interest starts at the sinotubular junction (**D**) and finishes at the slice just proximal to the origin of the brachiocephalic artery (**E**). The width of the volume of interest is increased to the maximum ascending aortic diameter + 4 mm (**F** + **H**). The ^18^F-sodium fluoride overlay is reinstated to ensure good coverage (**I**). The ascending aortic AMA, and volume are calculated (**I**). The aortic arch volume of interest is drawn with the same method as the ascending aorta, starting with the slice immediately distal to the ascending aortic volume of interest (**J**), and finishing with the slice after the origin of the left subclavian artery (**K**). The width of the aortic arch volume of interest is increased to the maximal arch dimeter + 4 mm (**M** + **N**). The ^18^F-sodium fluoride overlay is reinstated to check good coverage and calculate the aortic arch AMA and volume (**N**). (**O**) Provides the formula for calculating overall AMA, whilst (**P**) uses the values in the current case to provide a working example of AMA calculation

We modified the recently published technique for assessing global ^18^F-NaF uptake across the coronary arteries^22^,^23^ for use in the ascending aorta and aortic arch. Aortic ^18^F-NaF activity was measured within volumes of interest created around the aorta using a centreline function in a multiplanar reconstruction viewer (Figure 2). The final diameter of the ROI around the aorta was equal to the maximal luminal diameter of the aorta of that section plus 4 mm (the approximate spatial resolution of PET). This margin of error can be consistently drawn and was added because the spatial resolution of PET is limited, PET and CT may be misregistered, and tracer uptake is frequently highest around the outer perimeter of the vessel. The ascending aortic volume of interest started at the sinotubular junction and finished immediately proximal to the junction with the brachiocephalic artery. The aortic arch volume of interest started at the junction with the brachiocephalic artery and finished immediately distal to the junction with the left subclavian artery. The descending aorta was not quantified during this analysis due to overspill of ^18^F-NaF uptake originating from the adjacent thoracic spine.

Aortic microcalcification activity (AMA) represents the ratio of aortic activity to background radiotracer activity. Aortic activity is calculated by taking the cumulative voxel intensity in the aortic volumes of interest and dividing by the volume in cm^3^, to give aortic intensity per cm^3^. The background radiotracer activity is similarly calculated by dividing the cumulative radiotracer activity in the two 2-cm^3^ atrial volumes of interest, and dividing by the volume, giving background voxel intensity per cm^3^ (Figure 2). AMA is calculated by dividing aortic intensity per cm^3^ by background intensity per cm^3^ as a unitless number. Contamination from the sternum or clavicular bones was excluded by applying an upper voxel intensity limit to the AMA. This threshold is set at the SUVmax in a volume of interest out-with the sternum, excluding all values above it in calculations of AMA.

#### Whole vessel standardised uptake values and tissue to background ratios

Established methods for calculating whole vessel SUVmean and SUVmax were applied using methodology for the ascending aorta and aortic arch described previously.^12^,^24^ Briefly, on adjacent axial images, A series of 2-D regions of interest were drawn around the aorta on adjacent 3-mm slices beginning where the right pulmonary artery is first visible, finishing at the last slice in which the aortic arch is visible. The average SUVmean and SUVmax over all regions of interest (typically between 30 and 40 slices) were calculated (Figure 1). Tissue to background ratios (TBRs) were also calculated for each region of interest - performed by dividing SUVmean and SUVmax values by blood pool activity (TBRmean and TBRmax, respectively). TBR values were similarly averaged over all regions of interest for whole vessel TBRmean and TBRmax.

#### Most diseased segment standardized uptake values and tissue to background ratios

As described previously, using the same regions of interest drawn in whole vessel analysis, the most diseased segment approach considers only the three consecutive regions of interest with the highest mean (SUV_MDSmean_ and TBR_MDSmean_) and max (SUV_MDSmax_ and TBR_MDSmax_) values and therefore represents uptake in the single most intense lesion.^13^,^25^

### Observer Repeatability and Scan-Rescan Reproducibility

All baseline scans were interpreted by two trained observers (AF and ML) using all techniques described above (AMA, whole vessel analysis, most diseased segment). The 20 repeat scans were analysed for all methods by one of the trained observers (AF or ML), blinded to the original results, in a random order and more than 4 weeks after the first analysis of the baseline scans to minimise recall bias.

### Time Efficiency Analysis

In 10 randomly selected cases, the time taken to conduct each method (whole vessel analysis, most diseased segment and AMA) were recorded separately. The time taken to measure blood pool activity was excluded from the analysis as this is common to all techniques.

### Clinical Correlation

Framingham stroke risk score and Revised Framingham stroke risk score are validated risk scores for predicting the 10-year risk of stroke.^15^,^26^ Framingham risk score for hard coronary heart disease and American College of Cardiology/American Heart Association Atherosclerotic Cardiovascular Disease (ACC/AHA ASCVD) score are validated risk scores for predicting the 10-year risk of coronary events and cardiovascular events respectively.^27^,^28^ Each of these scores were calculated for each participant and the correlation with PET assessments of aortic ^18^F-NaF activity investigated.

### Statistical Analysis

All statistical analyses were performed in the open-source statistical software package R (V4.0.2). Continuous variables with normal distribution were presented as mean ± standard deviation, whereas non-normally distributed variables were presented as median [interquartile range]. Categorical variables were presented as number (percentage). Intra- and inter-observer variability as well as scan-rescan reproducibility were assessed using for each ^18^F-NaF aortic uptake method using mean error, 95% limits of agreement, coefficient of reproducibility, intraclass correlation coefficient and Bland-Altman plots.^29^ Associations between clinical risk scores and PET uptake methods were evaluated as a continuous variable (Pearson’s correlation coefficient). Statistical significance was taken as a two-sided *P*<0.05.

## Results

**Figure 3:**
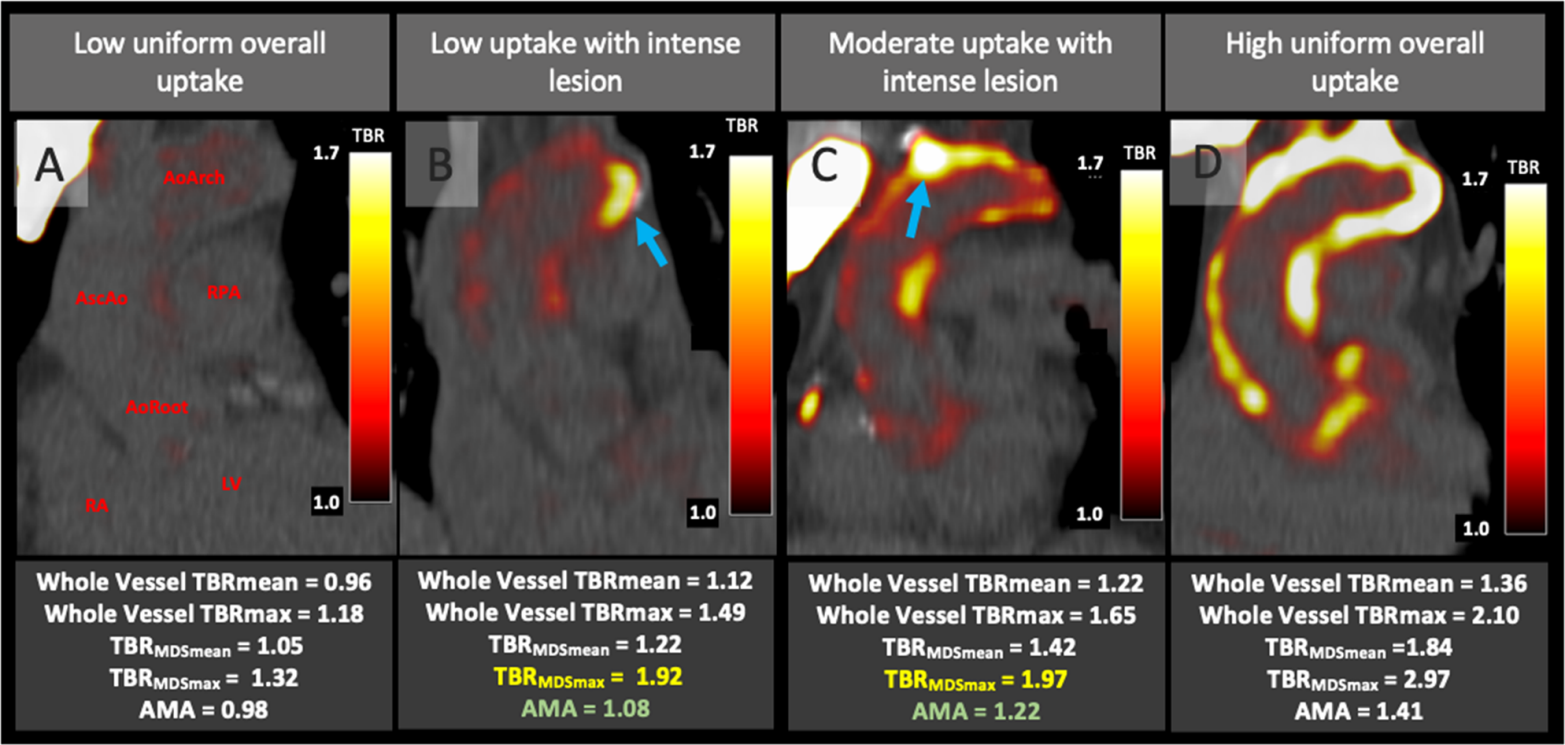
Hybrid ^18^F-sodium fluoride positron emission tomography and computed tomography coronal images of the ascending aorta and arch in four patients with varying patterns and intensity of aortic wall ^18^F-sodium fluoride activity: (**A**) Homogenously low activity across the ascending aorta and arch; (**B**) Generally low activity with a single high intensity lesion (blue arrow); (**C**) Moderate activity with a high intensity lesion (blue arrow); (**D**) High and intense activity throughout ascending aorta and arch. Note that (**B**) and (**C**) have similar values for most diseased segment maximum tissue to background ratio (highlighted in yellow) despite substantially different overall activity (aortic microcalcification activity values highlighted in green). *AscAo*, ascending aorta; *AMA*, aortic microcalcification activity; *AoArch*, aortic arch; *AoRoot*, aortic root; *MDS*, most diseased segment; *LV*, left ventricle; *RA*, right atrium; *RPA*, pulmonary artery; *SUV*, standardised uptake measurement; *TBR*, tissue to background ratio

^18^F-Sodium fluoride uptake was present in the ascending aorta and aortic arch of all twenty patients (Table 1). Uptake was seen in the aortic wall, although the pattern and degree of uptake varied markedly between patients (Figure 3). The AMA method was nearly 5 times quicker to perform than TBR analyses (3.4±0.5 versus 15.1±1.7 min, *P*<0.0001)

**Table 1:** Patient characteristics

Characteristic	Mean ± Standard Deviation or Number (%)
Age (years)	70±7
Female sex	3 (15%)
Type II diabetes mellitus	2 (10%)
Normal estimated glomerular filtration rate*	17 (85%)
Body-mass Index (kg/m^2^)	27±4
Smoking status	
Current	3 (15%)
Ex-smoker	14 (70%)
Never	3 (15%)
Hypertension	14 (70%)
Hypercholesterolaemia	20 (100%)
Previous myocardial infarction	13 (65%)
Previous stroke or transient ischaemic attack	1 (5%)
Previous revascularisation	
Coronary artery bypass graft	9 (45%)
Coronary stenting	13 (65%)
Medication	
Statin	20 (100%)
Beta-blocker	9 (45%)
Angiotensin-converting enzyme inhibitor	17 (85%)
Aspirin	20 (100%)
Left ventricular hypertrophy on electrocardiogram	0 (0%)

mean ± standard deviation; n (%)

*>60 mL/min/1.73 m^2^

### Intra-observer Repeatability of Aortic Microcalcification Activity

The AMA values ranged from 0.91 to 1.51 with a mean of 1.08±0.14. The intra-observer repeatability was excellent (intraclass correlation coefficient 0.98), with mean error 0.00, 95% limits of agreement of − 0.06 to 0.06, and coefficient of repeatability of 0.05. These results are similar to intra-observer repeatability for whole vessel and most diseased segment methods (Table 2, Supplementary Figure 1).

**Table 2:** Scan-rescan reproducibility, inter- and intra-observer reliability for whole vessel, most diseased segment and aortic microcalcification activity techniques

	Range	Mean	Mean Error (95% LOA)	Coefficient of Repeatability (% of mean)	Intraclass Correlation Coefficient
Aortic microcalcification activity	0.91 to 1.51	1.08±0.14			
Intra-observer			0.00 (− 0.06 to 0.06)	0.05 (4%)	0.98
Inter-observer			0.01 (− 0.05 to 0.07)	0.08 (6%)	0.97
Scan–rescan			− 0.00 (− 0.13 to 0.13)	0.11 (10%)	0.86
Whole vessel TBRmean	0.8 to 1.7	1.06±0.17			
Intra-observer			0.00 (− 0.03 to 0.03)	0.03 (3%)	0.99
Inter-observer			− 0.01 (− 0.16 to 0.15)	0.13 (12%)	0.87
Scan–rescan			0.01 (− 0.16 to 0.17)	0.17 (16%)	0.84
Whole vessel TBRmax	1.0 to 2.6	1.42±0.33			
Intra-observer			0.00 (− 0.03 to 0.04)	0.04 (3%)	0.99
Inter-observer			− 0.02 (− 0.24 to 0.20)	0.17 (12%)	0.93
Scan–rescan			0.03 (− 0.26 to 0.33)	0.33 (23%)	0.86
Most diseased segment TBR_MDSmean_	0.97 to 2.14	1.21±0.25			
Intra-observer			0.01 (− 0.04 to 0.05)	0.05 (4%)	0.99
Inter-observer			0.06 (− 0.14 to 0.26)	0.26 (21%)	0.94
Scan–rescan			0.02 (− 0.24 to 0.29)	0.30 (25%)	0.83
Most diseased segment TBR_MDSmax_	1.18 to 3.30	1.75±0.44			
Intra-observer			0.00 (− 0.05 to 0.06)	0.06 (3%)	0.99
Inter-observer			0.07 (− 0.21 to 0.34)	0.34 (19%)	0.93
Scan–rescan			0.03 (− 0.34 to 0.40)	0.39 (22%)	0.90

*LOA*, limits of agreement; *MDS*, most diseased segment; *SD*, standard deviation; *SUV*, standardized uptake value; *TBR*, tissue to background ratio; *TBR*_*MDSmean*_, most diseased segment tissue to background ratio mean, *TBR*_*MDSmax*_ most diseased segment tissue to background ratio maximum

### Inter-observer Repeatability of Aortic Microcalcification Activity

The inter-observer repeatability was excellent (intraclass correlation coefficient 0.97) with a mean error of 0.01, narrow 95% limits of agreement of − 0.05 to 0.07, and a coefficient of repeatability of 0.08 (Table 2, Supplementary Figure 2). Again, similar inter-observer repeatabilities were seen for whole vessel and most diseased segment methods (Table 2, Supplementary Figure 2).

### Scan-Rescan Reproducibility of Aortic Microcalcification Activity

**Figure 4:**
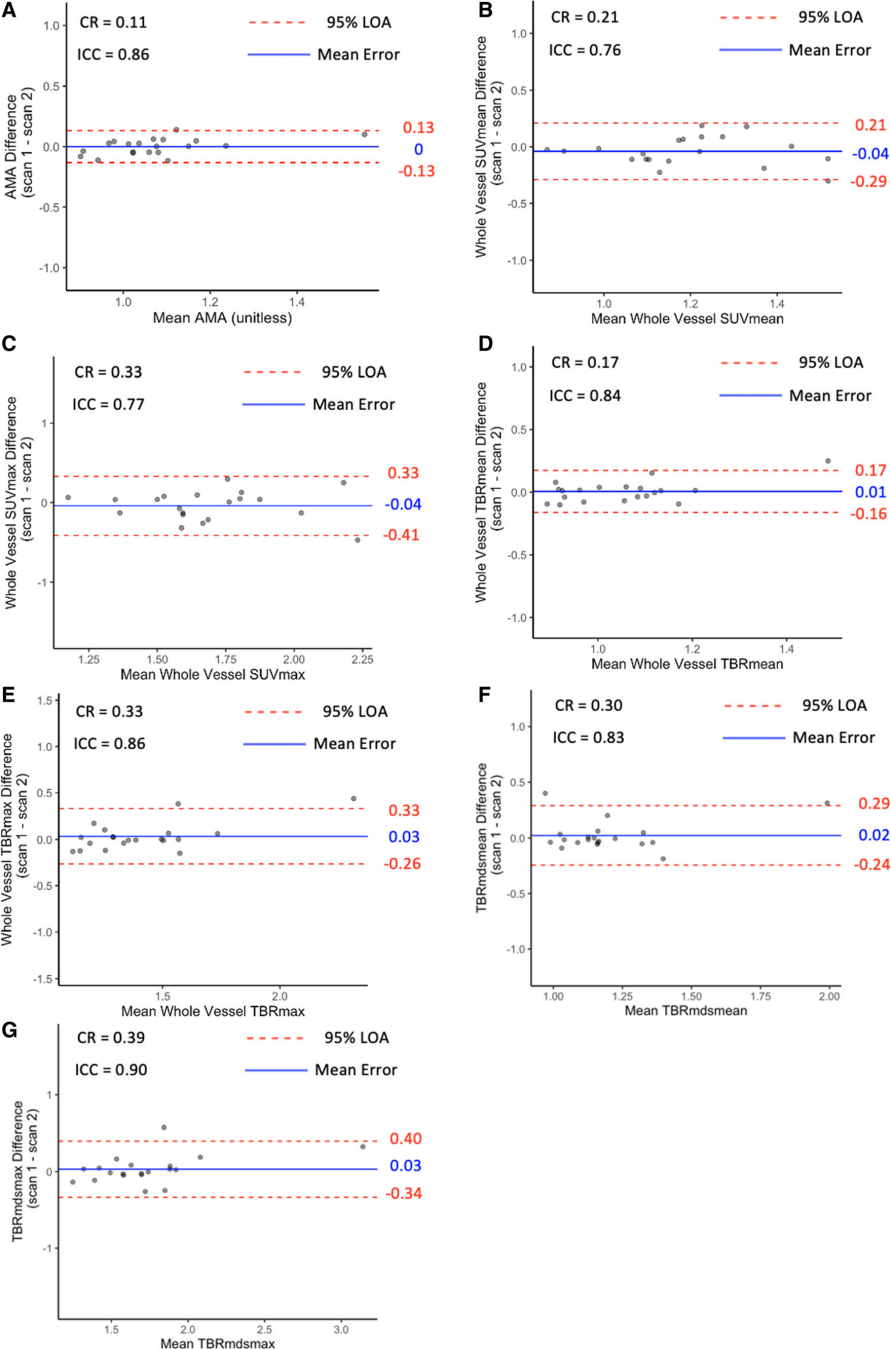
Scan-rescan reproducibility. Bland-Altmann plots with mean error (blue line) and 95% limits of agreement (red lines) for whole vessel standardized uptake value mean (**A**), standardized uptake value max (**B**), tissue to background ratio mean (**C**), tissue to background ratio max (**D**), most diseased segment tissue to background ratio mean (**E**) and tissue to background ratio maximum (**F**) and aortic microcalcificaion activity (**G**) methods. Y-axis limits are set to the method mean value of the method concerned. *AMA*, aortic microcalcification activity; *CR*, coefficient of reproducibility; *ICC*, intraclass correlation coefficient, *MDS*, most diseased segment; *LOA*, limits of agreement; *SD*, standard deviation; *TBR*, tissue to background ratio

The AMA method demonstrated very good scan-rescan reproducibility (intraclass correlation coefficient 0.86) with a minimal mean error of 0.00, narrow 95% limits of agreement of − 0.13 to 0.13, and a coefficient of reproducibility of 0.11 (Table 2, Figure 4). The scan-rescan reproducibility of AMA was similar to the whole vessel TBRmean (intraclass correlation coefficient 0.84) and TBRmax (intraclass correlation coefficient 0.86) as well as most diseased segment TBR_MDSmean_ (intraclass correlation coefficient 0.83) and TBR_MDSmax_ (intraclass correlation coefficient 0.90, Table 2 and Figure 4).

Scan-rescan reproducibility was unaffected by correction for either the time from radiotracer injection to PET imaging or aortic motion during the cardiac cycle (Table 3). The AMA scores were highly co-linear with the other methods, particularly TBRmax (Supplementary Figure 5).

**Table 3:** Influence of motion and background correction on aortic microcalcification activity scan-rescan reproducibility

	Range	Mean error (95% LOA)	Coefficient of reproducibility	Intraclass correlation coefficient
AMA	0.91 to 1.51	0.00 (− 0.13 to 0.13)	0.11 (10%)	0.86
AMA + time-delay correction	0.90 to 1.51	0.00 (− 0.13 to 0.14)	0.12 (11%)	0.85
AMA + motion correction	0.90 to 1.51	− 0.00 (− 0.14 to 0.13)	0.10 (9%)	0.85

*AMA*, aortic microcalcification activity; *LOA*, limits of agreement

### Correlation to Clinical Risk Score for Stroke

**Figure 5:**
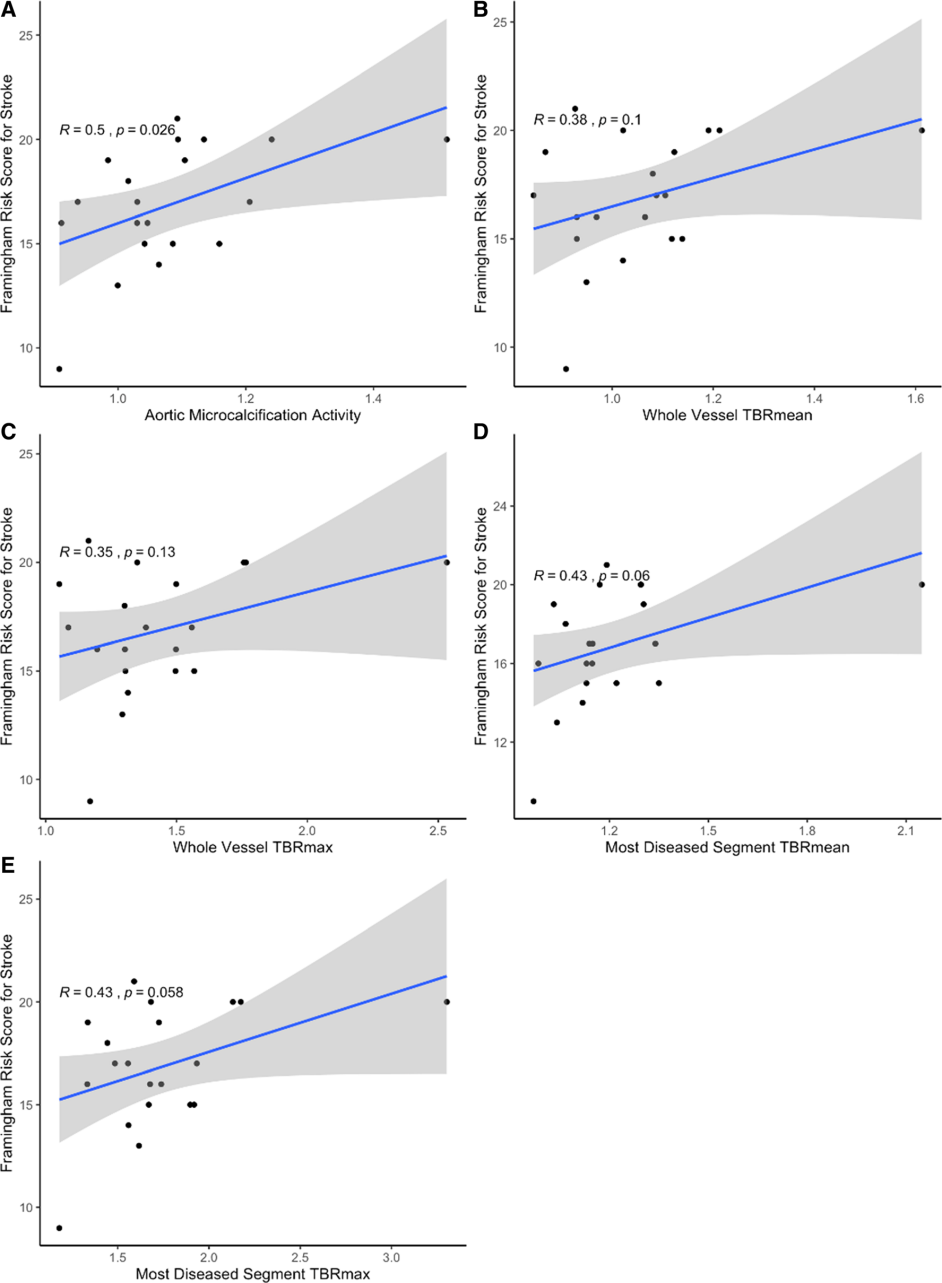
Scatterplots with Pearson’s correlation coefficients for the various methods of quantifying ^18^F-soidum fluoride uptake in the thoracic aorta compared with Framingham Risk Score for stroke in each patient. (**A**) Aortic macrocalcification activity (**B**) whole vessel TBRmean (**C**) whole vessel TBRmax (**D**) most diseased segment TBRmean (**E**) most diseased segment TBRmax. *AMA*, aortic microcalcification activity; *TBR* tissue to background ratio

There was a moderate and positive correlation between AMA and the Framingham stroke risk score (*R* = 0.50, *P* = 0.03, Figure 5), Revised Framingham stroke risk score (*R* = 0.44, *P* = 0.05) and Framingham risk Score for hard coronary (*R* = 0.44, *P* = 0.05, Table 4). Apparent weaker associations were observed between the other PET measures and Framingham stroke risk score (Figure 5) and revised Framingham stroke risk score (Table 4). As well as AMA, most diseased segment TBRmax demonstrated a moderate correlation with Framingham risk score for hard coronary heart disease (*R* = 0.48, *P* = 0.03). No risk scores correlated with the American College of Cardiology/American Heart Association atherosclerotic cardiovascular score (ACC/AHA ASCVD, Table 4).

**Table 4:** Correlation coefficients between aortic ^18^F-sodium fluoride quantification methods and clinical risk scores

	Framingham stroke risk score	Revised Framingham stroke risk score (10-year risk)	Framingham risk score for hard coronary events (10-year risk)	ACC/AHA atherosclerotic cardiovascular disease score (10-year risk)
Aortic microcalcification activity	R = 0.50*	R = 0.44*	R = 0.44*	*R* = 0.33
Whole vessel TBRmean	*R* = 0.38	*R* = 0.22	*R* = 0.21	*R* = 0.11
Whole vessel TBRmax	*R* = 0.35	*R* = 0.27	*R* = 0.32	*R* = 0.20
Most diseased segment TBRmean	*R* = 0.43	*R* = 0.41	*R* = 0.43	*R* = 0.29
Most diseased segment TBRmax	*R* = 0.43	*R* = 0.36	R = 0.48*	*R* = 0.33

*ACC*, American College of Cardiology; *AHA*, American heart association; *TBRmax*, maximum tissue to background ratio; *TBRmean*, mean tissue to background ratio

*P ≤ 0.05

## Discussion

Molecular imaging techniques are increasingly being used for investigating disease activity in the cardiovascular system. We describe a novel method, AMA, which quantifies ^18^F-NaF across both the ascending aorta and aortic arch, providing a measure of overall burden of disease activity in these vessels. We demonstrate this method as being highly reproducible and more time efficient than the whole vessel technique. Moreover, it can be performed with a non-contrast CT, and does not require advanced post-processing techniques, such as motion or time-delay correction, making it potentially more widely applicable. Finally, out of all methods assessed, AMA had the strongest correlation with Framingham stroke risk score and the revised Framingham stroke risk score. These results pave the way for future research investigating whether AMA holds advantages in terms of tracking disease progression and response to therapy as well as improving the prognostic performance of aortic PET.

There are several conceptual advantages to providing a more global assessment of ^18^F-NaF activity across the aorta than is provided by standard approaches. The TBRmax values in particular are based upon a small number of highly intense pixels and provide information about the peak intensity of a lesion. On the other hand, AMA incorporates both voxel intensity and volume, providing a global quantification of disease burden. These two approaches may have strengths under differing circumstances. For example, the whole vessel TBRmax and most diseased segment approaches may be more helpful in assessing diseases that are initiated by a threshold effect, such as plaque rupture or aortic dissection. In contrast, other diseases may be best captured by describing the overall burden of disease and AMA, such as aneurysm expansion or aortitis. However, theoretical application of such approaches does have limitations and depends on a number of factors. For example, we recently demonstrated that the summary measure of coronary microcalcification activity was the strongest predictor of future coronary events in patients with multivessel disease.^9^ This probably reflects the fact that plaque rupture commonly heals spontaneously without causing myocardial infarction and therefore a measure of overall disease activity is more powerful than focusing on a single lesion TBRmax. Whether a single intense lesion or overall disease activity better reflects risk of subsequent events in thoracic aortic disease, such as stroke in atherogenic patients or complications of thoracic aneurysm disease, remains to be seen.

In contrast to our findings in the coronary arteries,^18^ background and motion corrections make minimal difference to overall AMA reproducibility. The time-delay blood pool correction accounts for the different elimination rates between the coronary arteries and blood pool seen over time.^18^,^20^ However, the elimination rates for the aorta over the same periods are different to those seen in the coronaries (Supplemental Figure 3). The time-delay blood pool correction formula used in coronary microcalcification activity should, therefore, not be applied to the AMA measurements. Motion correction is necessary in assessing the uptake in the coronary arteries as they are relatively small vessels, with potential contamination from surrounding structures (e.g. mitral valve annular calcification), partial volume effects and marked movement throughout the cardiac cycle. The aorta, on the other hand, is a large and relatively stationary vessel, with little contamination from surrounding structures and reduced suseptability to potential partial volume effects, although these still may be present. Moreover, our technique for drawing AMA volumes of interest was standardised to 4 mm beyond the maximal lumen diameter, likely incorporating most aortic movement. This probably explains why motion correction had no effect on AMA values.

It is important to highlight some limitations to our study. Due to well documented problems with spinal contamination influencing accurate ^18^F-NaF assessment in the descending thoracic aorta, we chose to limit our AMA analysis to the ascending aorta and aortic arch. Importantly our AMA approach could also be applied to other tracers used to assess disease activity in the aorta (e.g. ^18^F-FDG or ^68^Ga-Dotatate), where such contamination is not an issue and where a global assessment of uptake might also include activity in the descending aorta. Although we have demonstrated the favourable efficiency, reproducibility and repeatability of AMA with positive correlations with clinical risk scores, whether or not AMA will improve the prediction of disease progression and cardiovascular events remains to be seen.

## New Knowledge Gained

Aortic microcalcification activity is a simple, repeatable and reproducible method for quantifying ^18^F-NaF uptake in the ascending aorta and arch that is significantly and substantially quicker to perform compared with alternative methods and correlates with validated cardiovascular risk scores.

## Conclusion

In conclusion, we have provided a detailed description of how to assess global ^18^F-NaF activity across both the ascending aorta and aortic arch using a time efficient approach that demonstrates highly favourable repeatability and reproducibility. Studies assessing the ability of AMA to track disease progression and response to therapy as well as predicting cardiovascular outcomes are now required to validate AMA as a novel biomarker of aortic disease.

## Electronic supplementary material

Below is the link to the electronic supplementary material.

Electronic supplementary material 1 (DOCX 824 kb)

Electronic supplementary material 2 (PPTX 1070 kb)

Electronic supplementary material 3 (M4A 3936 kb)

## Abbreviations

^18^F-NaF: Sodium fluoride
AMA: Aortic microcalcification activity
CT: computed tomography
PET: Positron emission tomography
SUV: standardised uptake value
TBR: Tissue to background ratio
TBR_MDS_: Most diseased segment tissue to background ratio
