# Supplementary material for: Quantifying microcalcification activity in the thoracic aorta
Source: J Nucl Cardiol. 2021 Jan 20;29(3):1372–85. doi: 10.1007/s12350-020-02458-w (PMC8497049; doi:10.1007/s12350-020-02458-w)
Supplement: Supplementary file 1 — Electronic supplementary material 1 (DOCX 824 kb) [file 12350_2020_2458_MOESM1_ESM.docx]

**Supplementary Table 1**

Interscan reproducibility, inter- and intra-observer reliability for whole vessel and most diseased segment standardised uptake values

|  | **Range** | **Mean**  **(SD)** | **Mean Error**  **(95% LOA)** | **Coefficient of Reproducibility/Repeatability** | **Interclass Correlation Coefficient** |
| --- | --- | --- | --- | --- | --- |

| **Whole Vessel SUVmean** | 0.8 to 1.5 | 1.17±0.18 |  |  |  |
| --- | --- | --- | --- | --- | --- |
| Intra-observer |  |  | 0.00  (-0.01 to 0.02) | 0.01 (1%) | 0.99 |
| Inter-observer |  |  | -0.04  (-0.17 to 0.08) | 0.14 (12%) | 0.91 |
| Scan-Rescan |  |  | -0.04  (-0.29 to 0.21) | 0.21 (18%) | 0.76 |
| **Whole Vessel SUVmax** | 1.2 to 2.4 | 1.66±0.27 |  |  |  |
| Intra-observer |  |  | 0.00  (-0.02 to 0.01) | 0.01 (1%) | 0.99 |
| Inter-observer |  |  | 0.00  (-0.02 to 0.03) | 0.03 (2%) | 0.99 |
| Scan-Rescan |  |  | -0.04  (-0.41 to 0.33) | 0.33 (20%) | 0.77 |

| **Most Diseased Segment SUV_mdsmean_** | 0.97 to 1.89 | 1.35±0.23 |  |  |  |
| --- | --- | --- | --- | --- | --- |
| Intra-observer |  |  | 0.00  (-0.03, 0.04) | 0.04 (3%) | 0.99 |
| Inter-observer |  |  | -0.03  (-0.09, 0.14) | 0.15 (11%) | 0.96 |
| Scan-Rescan |  |  | -0.02  (-0.38, 0.34) | 0.36 (27%) | 0.71 |
| **Most Diseased Segment SUV_mdsmax_** | 1.31 to 2.91 | 1.93±0.38 |  |  |  |
| Intra-observer |  |  | 0.00  (-0.05, 0.05) | 0.05 (3%) | 0.99 |
| Inter-observer |  |  | 0.01  (-0.1, 0.15) | 0.18 (9%) | 0.98 |
| Scan-Rescan |  |  | -0.04  (-0.47, 0.39) | 0.44 (23%) | 0.84 |

AMA = aortic microcalcification activity, LOA = limits of agreement, max = maximum, MDS = most diseased segment, SD = standard deviation, SUV = standardized uptake value

**
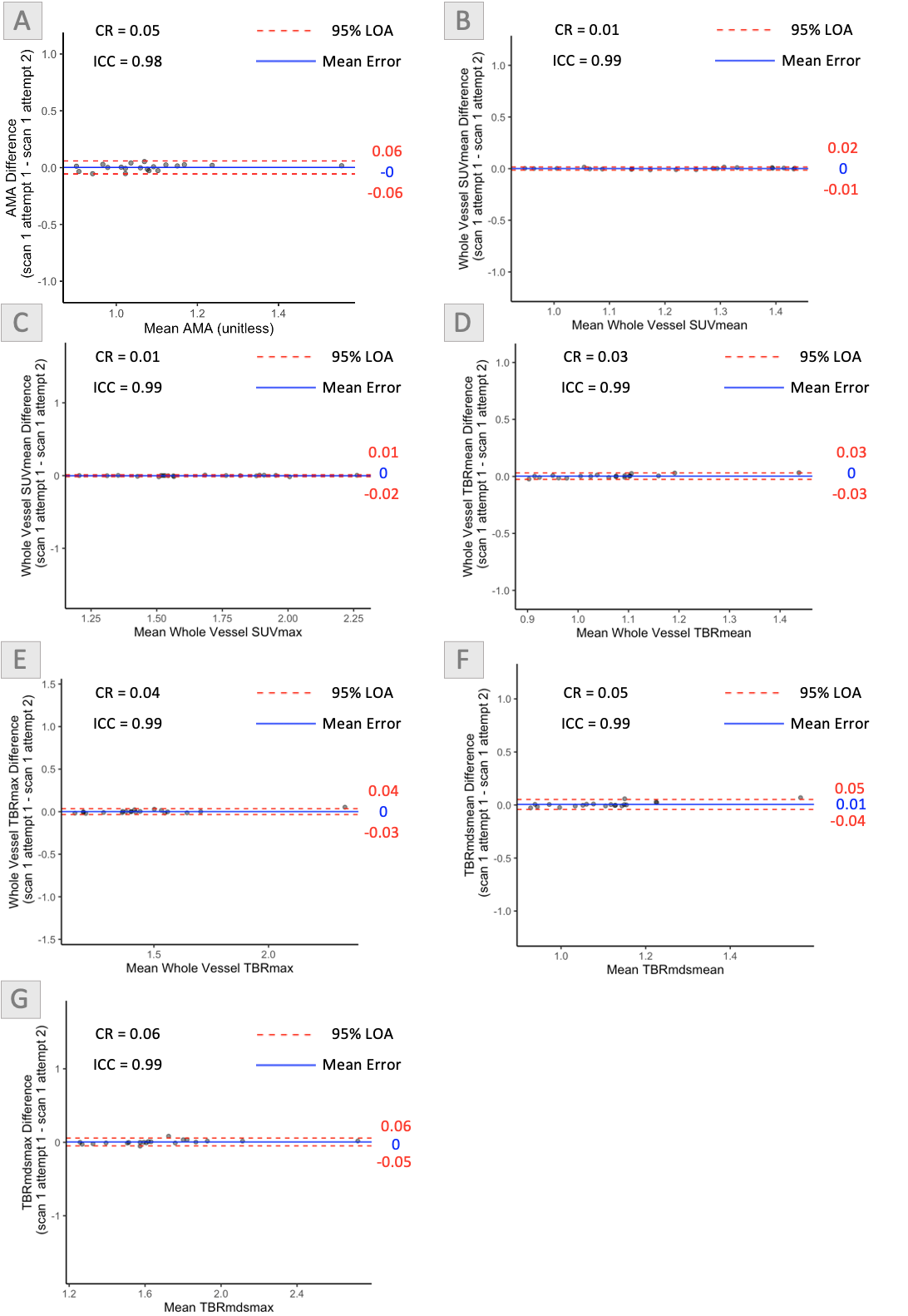
Supplemental Figure 1**

**Supplemental Figure 1**: Intra-observer repeatability. Bland-Altmann plots with mean error (blue line) and 95% limits of agreement (red lines) for whole vessel standardized uptake value mean (A), standardized uptake value max (B), tissue to background ratio mean (C), tissue to background ratio max (D), most diseased segment tissue to background ratio mean (E) and tissue to background ratio maximum (F) and aortic microcalcificaion activity (G) methods. Y-axis limits are set to the method mean value of the method concerned.

AMA = aortic microcalcification activity, CR = coefficient of reproducibility, ICC = intraclass correlation coefficient, MDS = most diseased segment, LOA = limits of agreement, SD = standard deviation, TBR = tissue to background ratio

**Supplemental Figure 2**

**
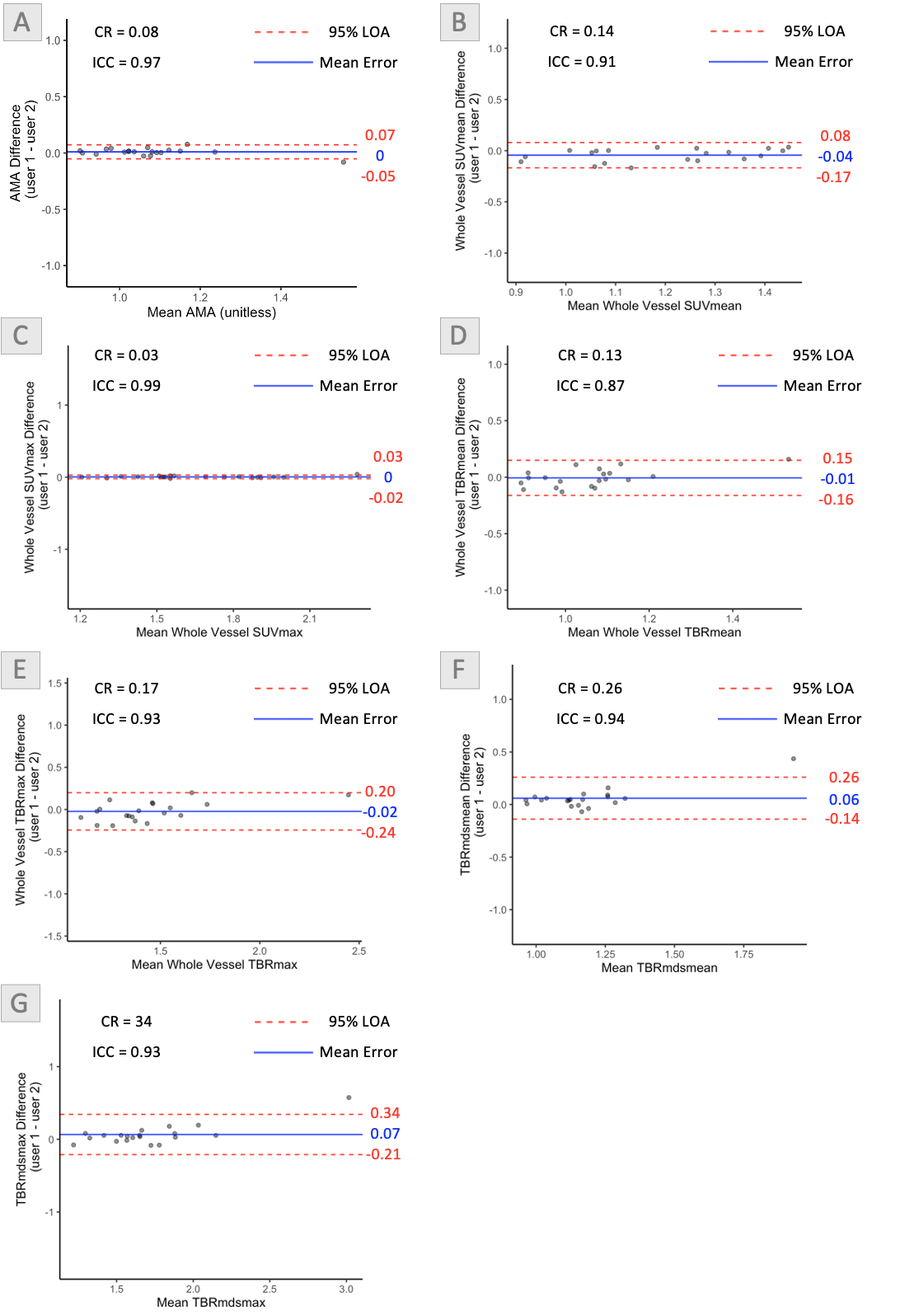
**

**Supplemental Figure 2**: Inter-observer reproducibility. Bland-Altmann plots with mean error (blue line) and 95% limits of agreement (red lines) for whole vessel standardized uptake value mean (A), standardized uptake value max (B), tissue to background ratio mean (C), tissue to background ratio max (D), most diseased segment tissue to background ratio mean (E) and tissue to background ratio maximum (F) and aortic microcalcificaion activity (G) methods. Y-axis limits are set to the method mean value of the method concerned.

AMA = aortic microcalcification activity, CR = coefficient of reproducibility, ICC = intraclass correlation coefficient, MDS = most diseased segment, LOA = limits of agreement, SD = standard deviation, TBR = tissue to background ratio

**Supplemental Figure 3**

**
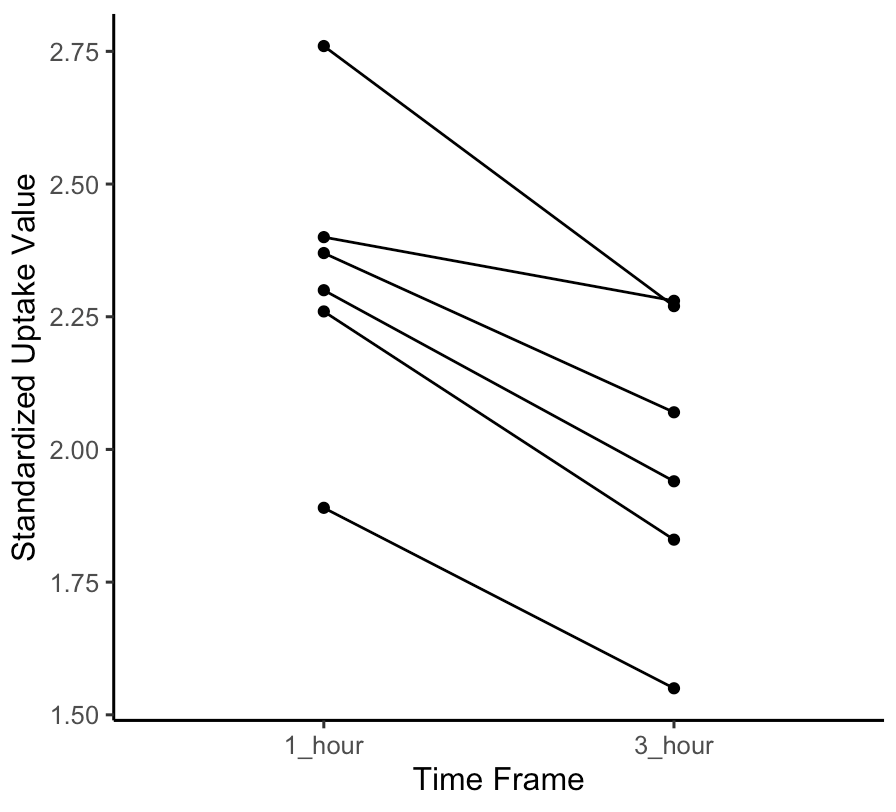
**

**Supplemental Figure 3**: The standardized uptake values in the aorta between at 1 and 3 hours demonstrates a falls in values for each patient. This is different to the pattern seen in the coronary arteries where uptake does not fall over time.

**Supplementary Figure 4**

**
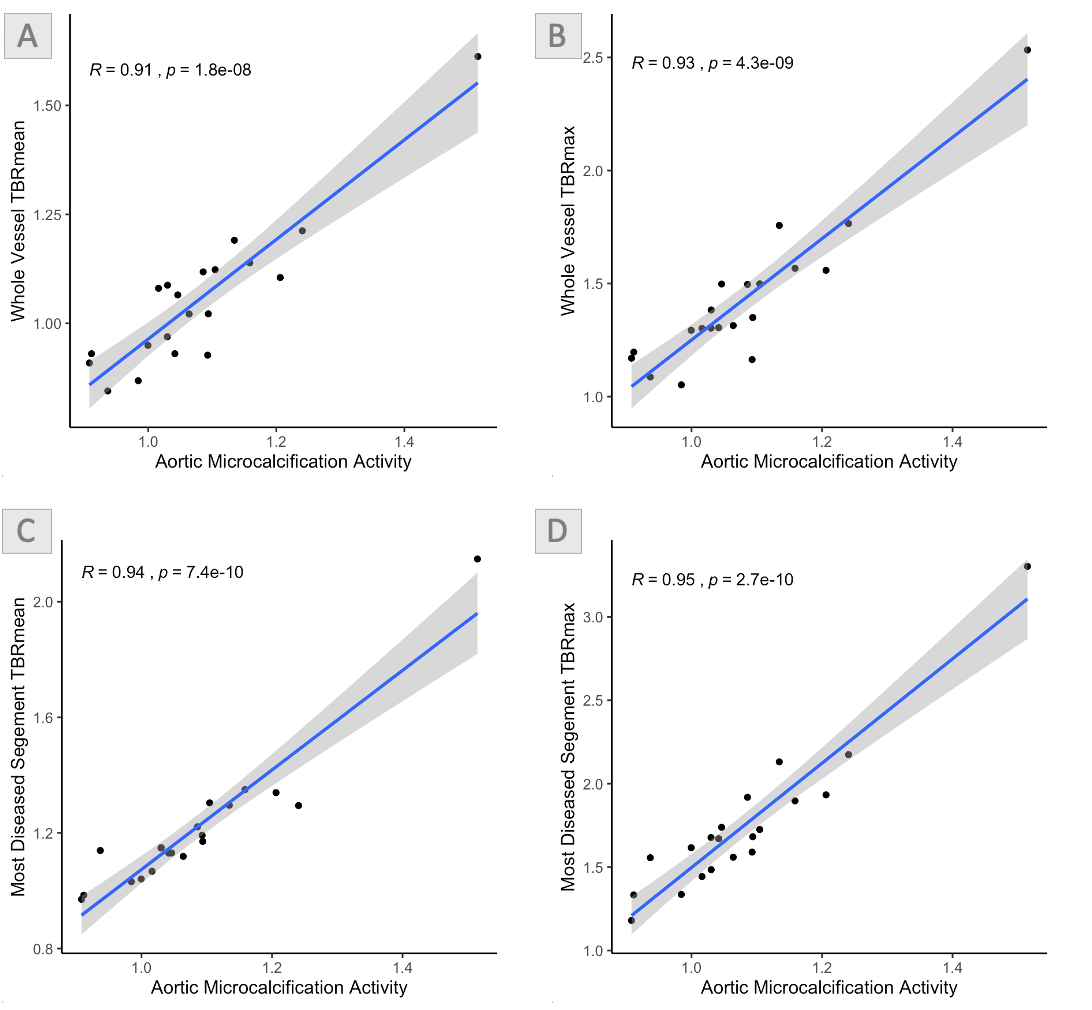
**

**Supplementary Figure 4:** Scatterplots for aortic microcalcification activity compared with other methods for calculating ^18^F-sodium fluoride uptake in the thoracic aorta demonstrating a largely co-linear relationship. (A) whole vessel TBRmean (B) whole vessel TBRmax (C) most diseased segment TBRmean (D) most diseased segment TBRmax. AMA = aortic microcalcification activity, TBR = tissue to background ratio.
