# Supplementary material for: Quantifying microcalcification activity in the thoracic aorta
Source: J Nucl Cardiol. 2021 Jan 20;29(3):1372–85. doi: 10.1007/s12350-020-02458-w (PMC8497049; doi:10.1007/s12350-020-02458-w)
Supplement: Supplementary file 2 — Electronic supplementary material 2 (PPTX 1070 kb) [file 12350_2020_2458_MOESM2_ESM.pptx]

## Slide 1
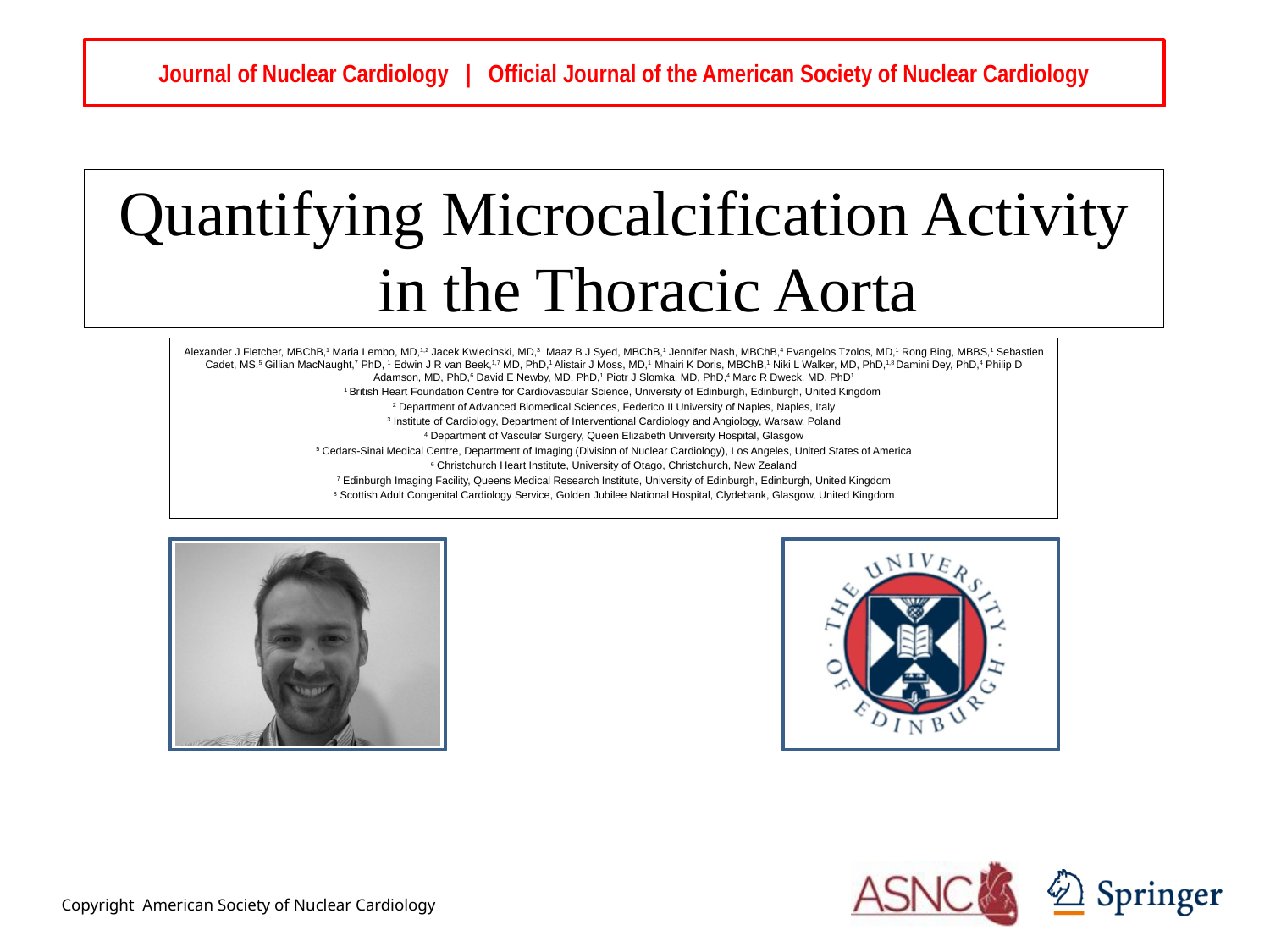

Journal of Nuclear Cardiology | Official Journal of the American Society of Nuclear Cardiology
# Quantifying Microcalcification Activity in the Thoracic Aorta
Alexander J Fletcher, MBChB,1 Maria Lembo, MD,1,2 Jacek Kwiecinski, MD,3 Maaz B J Syed, MBChB,1 Jennifer Nash, MBChB,4 Evangelos Tzolos, MD,1 Rong Bing, MBBS,1 Sebastien Cadet, MS,5 Gillian MacNaught,7 PhD, 1 Edwin J R van Beek,1,7 MD, PhD,1 Alistair J Moss, MD,1 Mhairi K Doris, MBChB,1 Niki L Walker, MD, PhD,1,8 Damini Dey, PhD,4 Philip D Adamson, MD, PhD,6 David E Newby, MD, PhD,1 Piotr J Slomka, MD, PhD,4 Marc R Dweck, MD, PhD1
1 British Heart Foundation Centre for Cardiovascular Science, University of Edinburgh, Edinburgh, United Kingdom
2 Department of Advanced Biomedical Sciences, Federico II University of Naples, Naples, Italy
3 Institute of Cardiology, Department of Interventional Cardiology and Angiology, Warsaw, Poland
4 Department of Vascular Surgery, Queen Elizabeth University Hospital, Glasgow
5 Cedars-Sinai Medical Centre, Department of Imaging (Division of Nuclear Cardiology), Los Angeles, United States of America
6 Christchurch Heart Institute, University of Otago, Christchurch, New Zealand
7 Edinburgh Imaging Facility, Queens Medical Research Institute, University of Edinburgh, Edinburgh, United Kingdom
8 Scottish Adult Congenital Cardiology Service, Golden Jubilee National Hospital, Clydebank, Glasgow, United Kingdom
Head shot of author
required
Institution
Picture/Logo
Optional
Copyright American Society of Nuclear Cardiology

## Slide 2
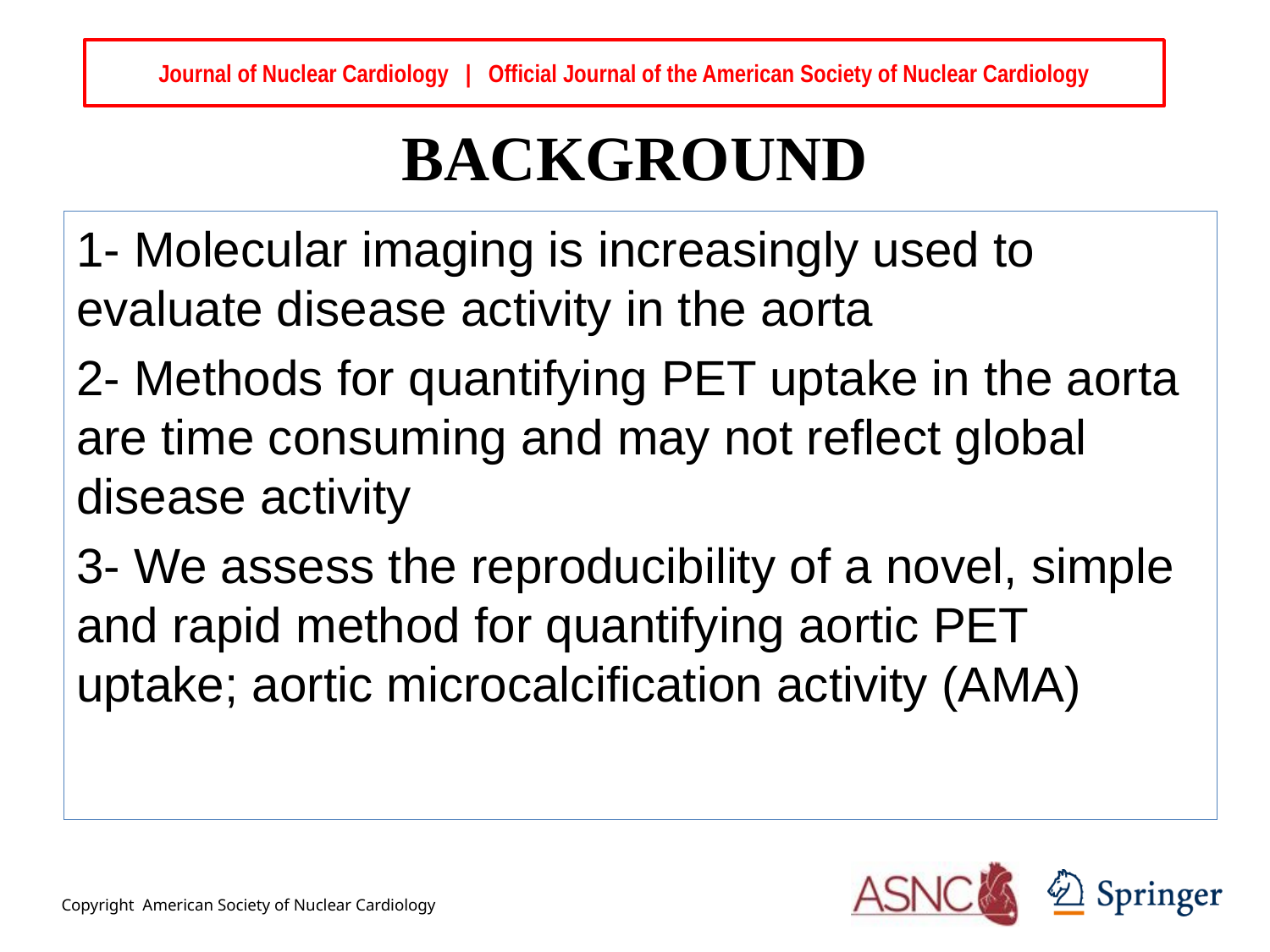

Journal of Nuclear Cardiology | Official Journal of the American Society of Nuclear Cardiology
# BACKGROUND
1- Molecular imaging is increasingly used to evaluate disease activity in the aorta
2- Methods for quantifying PET uptake in the aorta are time consuming and may not reflect global disease activity
3- We assess the reproducibility of a novel, simple and rapid method for quantifying aortic PET uptake; aortic microcalcification activity (AMA)
Copyright American Society of Nuclear Cardiology

## Slide 3
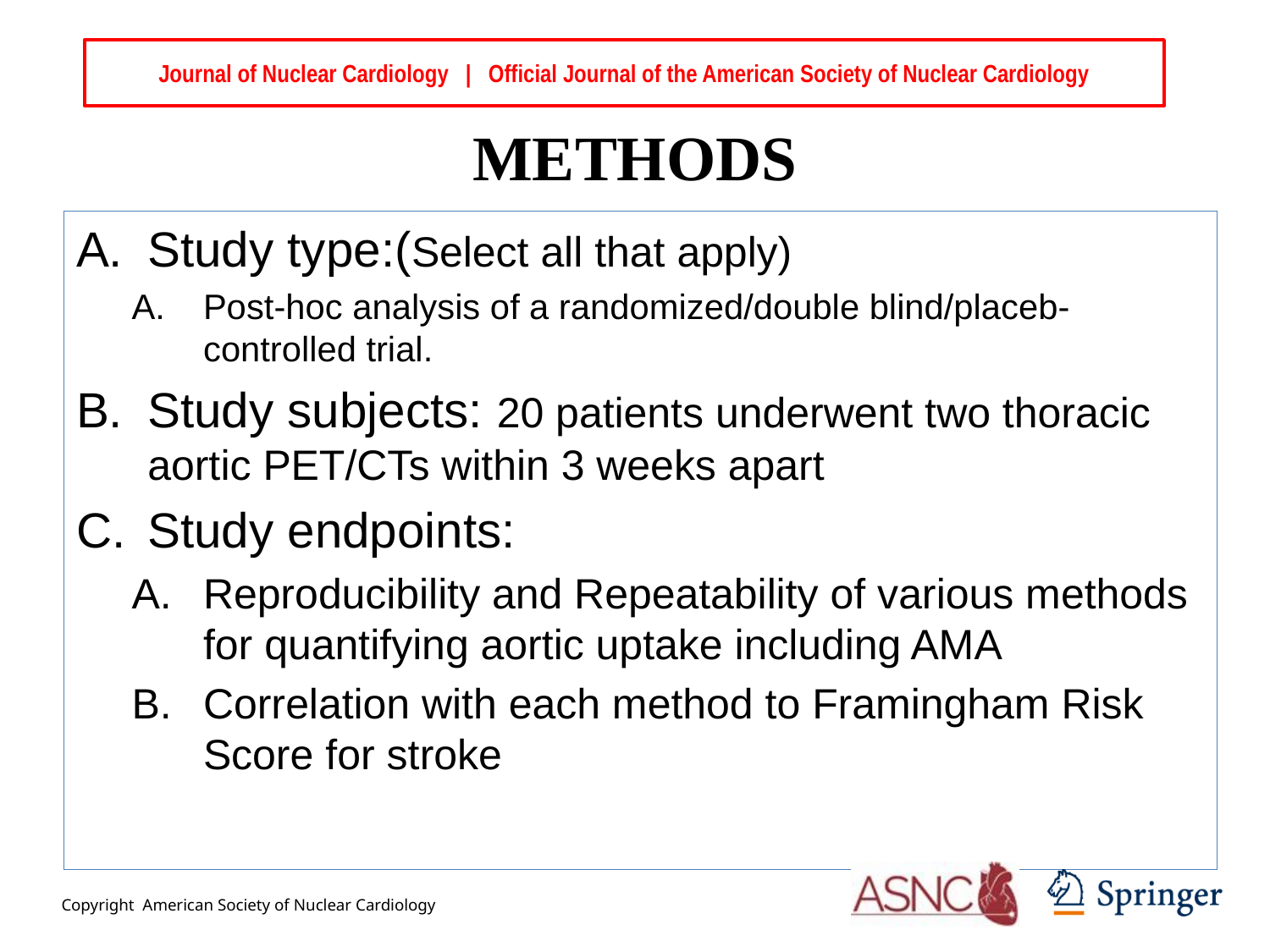

Journal of Nuclear Cardiology | Official Journal of the American Society of Nuclear Cardiology
# METHODS
Study type:(Select all that apply)
Post-hoc analysis of a randomized/double blind/placeb- controlled trial.
Study subjects: 20 patients underwent two thoracic aortic PET/CTs within 3 weeks apart
Study endpoints:
Reproducibility and Repeatability of various methods for quantifying aortic uptake including AMA
Correlation with each method to Framingham Risk Score for stroke
Copyright American Society of Nuclear Cardiology

## Slide 4
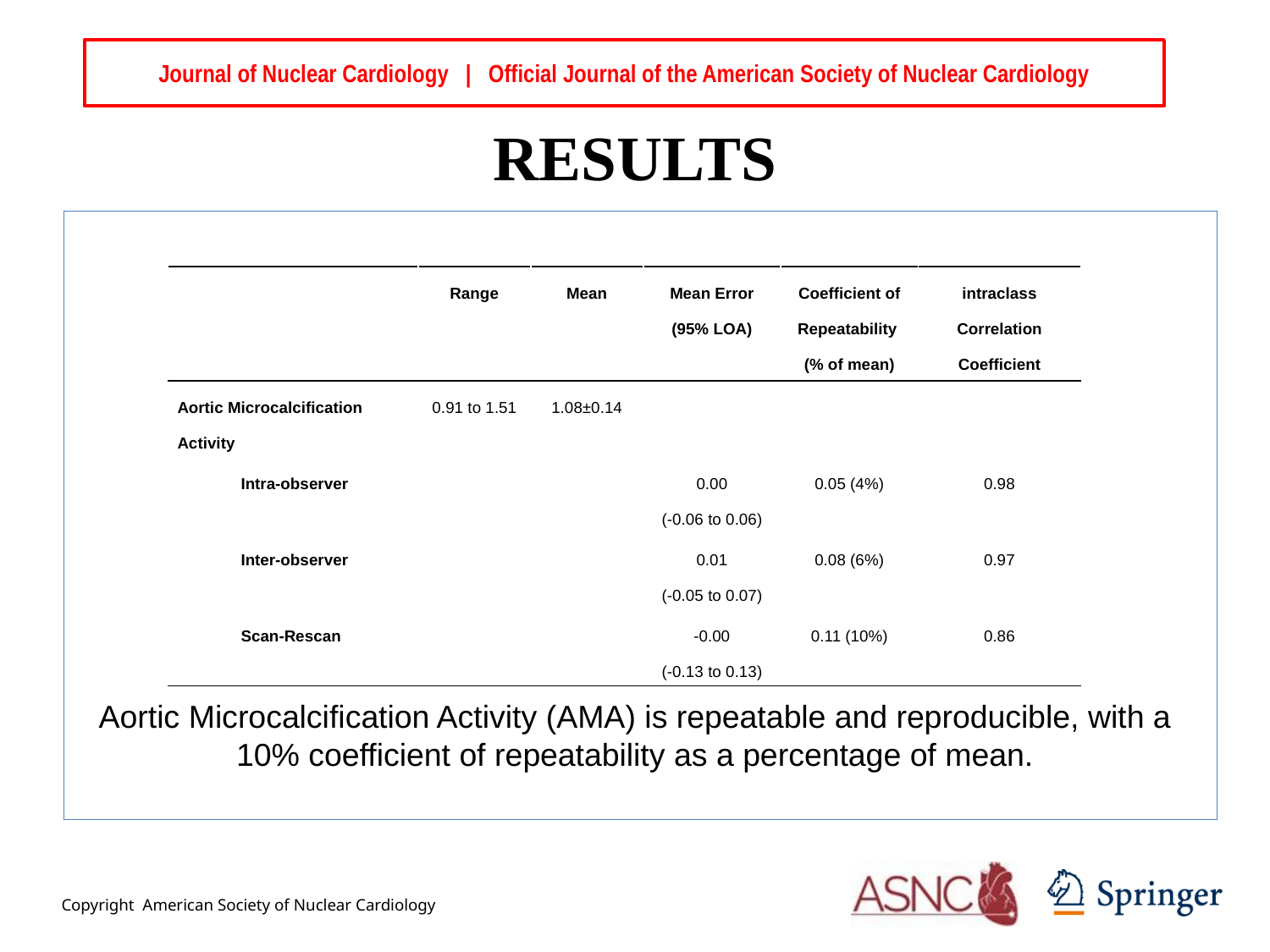

Journal of Nuclear Cardiology | Official Journal of the American Society of Nuclear Cardiology
# RESULTS
| | Range | Mean | Mean Error (95% LOA) | Coefficient of Repeatability (% of mean) | intraclass Correlation Coefficient |
| --- | --- | --- | --- | --- | --- |
| Aortic Microcalcification Activity | 0.91 to 1.51 | 1.08±0.14 | | | |
| Intra-observer | | | 0.00 (-0.06 to 0.06) | 0.05 (4%) | 0.98 |
| Inter-observer | | | 0.01 (-0.05 to 0.07) | 0.08 (6%) | 0.97 |
| Scan-Rescan | | | -0.00 (-0.13 to 0.13) | 0.11 (10%) | 0.86 |
Copyright American Society of Nuclear Cardiology

## Slide 5
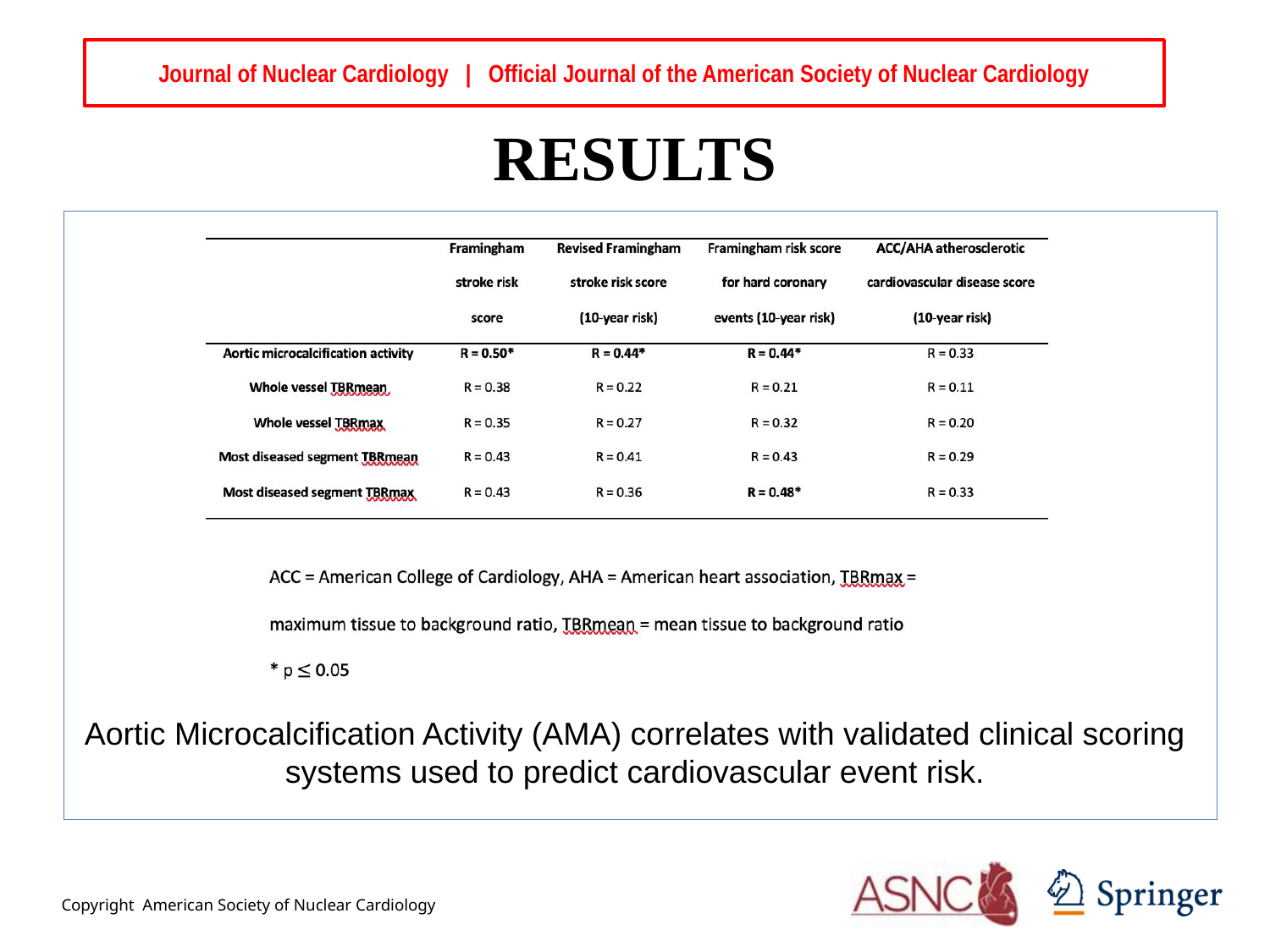

Journal of Nuclear Cardiology | Official Journal of the American Society of Nuclear Cardiology
# RESULTS
Aortic Microcalcification Activity (AMA) correlates with validated clinical scoring systems used to predict cardiovascular event risk.
Copyright American Society of Nuclear Cardiology

## Slide 6
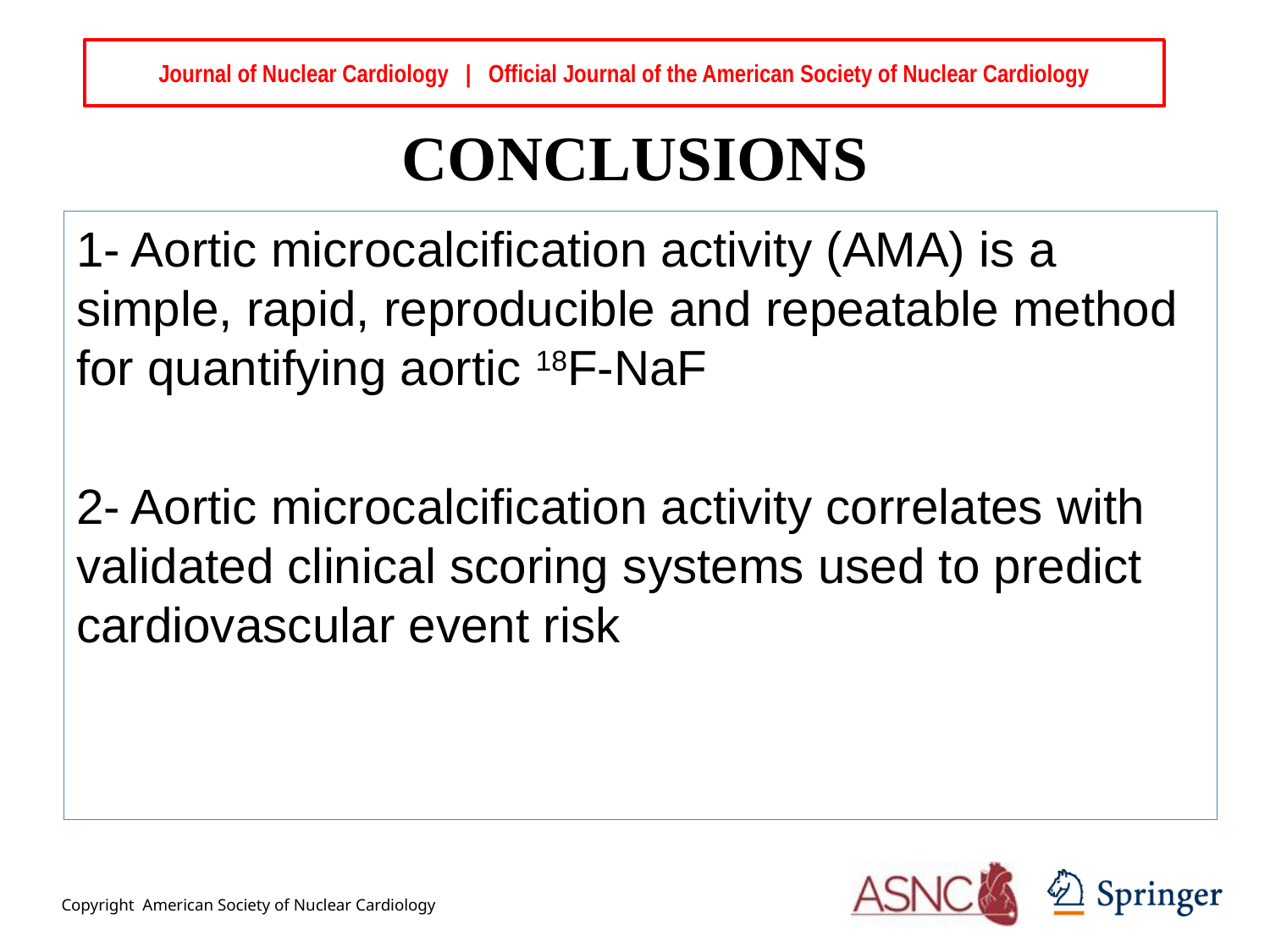

Journal of Nuclear Cardiology | Official Journal of the American Society of Nuclear Cardiology
# CONCLUSIONS
1- Aortic microcalcification activity (AMA) is a simple, rapid, reproducible and repeatable method for quantifying aortic 18F-NaF
2- Aortic microcalcification activity correlates with validated clinical scoring systems used to predict cardiovascular event risk
Copyright American Society of Nuclear Cardiology
